# A Treatise of the Diseases of the Nervous System

**Published:** 1886-08

**Authors:** 


**A Treatise on the Diseases of the Nervous System.** By WILLIAM A. HAMMOND, M. D., Surgeon-General United States Army, (retired list,) Professor of the Diseases of the Mind and Nervous System, in the New York Post-Graduate School and Hospital, etc. Eighth edition, with corrections and additions. New York: D. Appleton & Co. 1886.

This volume has been received by the profession "to an extent beyond that ever given to any other work of like scope and objects published in any part of the world." The present edition contains a section on "Certain Obscure Diseases of the Nervous System," is thoroughly revised throughout, and several changes made, thereby increasing greatly its usefulness. We can add, it is one of the most complete works on this subject; it throws light on many points made more obscure by many writers on this subject.

